# A novel splicing site *IRP1* somatic mutation in a patient with pheochromocytoma and *JAK2*^V617F^ positive polycythemia vera: a case report

**DOI:** 10.1186/s12885-018-4127-x

**Published:** 2018-03-13

**Authors:** Ying Pang, Garima Gupta, Chunzhang Yang, Herui Wang, Thanh-Truc Huynh, Ziedulla Abdullaev, Svetlana D. Pack, Melanie J. Percy, Terence R. J. Lappin, Zhengping Zhuang, Karel Pacak

**Affiliations:** 1 Section on Medical Neuroendocrinology, Eunice Kennedy Shriver National Institute of Child Health and Human Development, National Institutes of Health, Bethesda, MD 20892 USA; 2 Neuro-Oncology Branch, Center for Cancer Research, National Cancer Institute, Bethesda, MD 20892 USA; 3 Laboratory of Pathology, National Cancer Institute, Bethesda, MD 20892 USA; 4 Department of Haematology, Belfast City Hospital, Belfast, Northern Ireland BT9 7AB UK; 5 Centre for Cancer Research and Cell Biology, Queen’s University, Belfast, Northern Ireland BT9 7AB UK

**Keywords:** Pheochromocytoma, Polycythemia, Splicing site, IRP

## Abstract

**Background:**

The role of the hypoxia signaling pathway in the pathogenesis of pheochromocytoma/paraganglioma (PPGL)-polycythemia syndrome has been elucidated. Novel somatic mutations in *hypoxia-inducible factor type 2A* (*HIF2A*) and germline mutations in *prolyl hydroxylase type 1 and type 2* (*PHD1* and *PHD2*) have been identified to cause upregulation of the hypoxia signaling pathway and its target genes including *erythropoietin (EPO)* and its receptor *(EPOR)*. However, in a minority of patients presenting with this syndrome, the genetics and molecular pathogenesis remain unexplained. The aim of the present study was to uncover novel genetic causes of PPGL-polycythemia syndrome.

**Case presentation:**

A female presented with a history of *JAK2*^V617F^ positive PV, diagnosed in 2007, and right adrenal pheochromocytoma diagnosed and resected in 2011. Her polycythemia symptoms and hematocrit levels continued to worsen from 2007 to 2011, with an increased frequency of phlebotomies. Postoperatively, until early 2013, her hematocrit levels remained normalized. Following this, the hematocrit levels ranged between 46.4 and 48.9% [35–45%]. Tumor tissue from the patient was further tested for mutations in genes related to upregulation of the hypoxia signaling pathway including *iron regulatory protein 1* (*IRP1*), which is a known regulator of HIF-2α mRNA translation. Functional studies were performed to investigate the consequences of these mutations, especially their effect on the HIF signaling pathway and EPO. Indel mutations (c.267-1_267delGGinsTA) were discovered at the exon 3 splicing site of *IRP1*. Minigene construct and splicing site analysis showed that the mutation led to a new splicing site and a frameshift mutation of *IRP1*, which caused a truncated protein. Fluorescence in situ hybridization analysis demonstrated heterozygous *IRP1* deletions in tumor cells. Immunohistochemistry results confirmed the truncated IRP1 and overexpressed HIF-2α, EPO and EPOR in tumor cells.

**Conclusions:**

This is the first report which provides direct molecular genetic evidence of association between a somatic *IRP1* loss-of-function mutation and PHEO and secondary polycythemia. In patients diagnosed with PHEO/PGL and polycythemia with negative genetic testing for mutations in *HIF2A, PHD1/2*, and *VHL*, *IRP1* should be considered as a candidate gene.

## Background

Polycythemia or absolute erythrocytosis is defined by an increase in red blood cell mass, reflected by elevated hemoglobin and hematocrit levels in patients with normal plasma volumes [1]. Primary polycythemia occurs due to somatic and germline mutations in erythroid, granulocytic and megakaryocytic progenitors with hypersensitive erythropoietin receptors (EPOR), leading to their clonal myeloproliferation with plasma erythropoietin (EPO) level below or in the normal range. Within this category, polycythemia vera is the most common *BCR-ABL1*-negative myeloproliferative neoplasm [2, 3]. Most cases of polycythemia vera are due to gain-of-function *JAK2* (Janus Kinase 2; 9p24) somatic mutations, over 95% of which are located in exon 14 (*JAK2*^V617F^) [2–5]. On the other hand, secondary polycythemia occurs due to high levels of circulating factors, usually EPO, which is a principal regulator of erythropoiesis [1]. In disorders due to genetic mutations of the hypoxia signaling pathway, there is both, aberrant production of EPO, a downstream target gene and increased sensitivity of erythroid progenitors to this hormone [6]. Until recently, Chuvash polycythemia, caused by germline R200W homozygous mutations in the *von Hippel-Lindau (VHL)* gene, was the only known form of congenital polycythemia to occur due to upregulation of the hypoxia signaling pathway [6–8]. Mutations in *prolyl hydroxylase domain (PHD) 1* and *2* and *hypoxia-inducible factor type 2A (HIF2A)* genes cause a syndromic presentation with polycythemia and erythropoietin secreting neuroendocrine malignancies. While previously, germline *PHD2* and *HIF2A* mutations were reported in rare cases of familial erythrocytosis, recent evidence elucidated the significance of the hypoxia signaling pathway in development of pheochromocytoma/paraganglioma (PPGL) [9–16].

PPGLs are rare catecholamine secreting neuroendocrine tumors derived either from chromaffin cells of the adrenal medulla or sympathetic and parasympathetic ganglia located outside of the adrenal gland. Patients with somatic *HIF2A* mutations, also referred to as Pacak-Zhuang syndrome, are typically females, diagnosed with polycythemia within the first decade of life, recurrent PPGLs, somatostatinomas and ocular abnormalities [9, 12, 17]. Patients with germline *PHD1* and *PHD2* mutations can develop polycythemia and recurrent PPGLs [13–15]. However, some patients with PPGL-polycythemia syndrome who lack identified mutations in the aforementioned genes remain as medical dilemmas, suggesting the existence of other, as yet undiscovered, contributing pathogenetic factors. Given a similar clinical phenotype, we screened for mutations in other unexplored regulators of HIF-2α, including *iron regulatory proteins* (*IRPs*), in one of our patients. IRP 1 and 2 are central regulators of cellular iron metabolism in mammalian cells, which posttranscriptionally bind to iron-responsive element (IRE) sequences in transcripts encoding iron metabolism proteins [18, 19]. Unlike IRP2, IRP1 is a bifunctional protein, which under iron-deficient conditions, binds to IREs to suppress protein translation and under iron-replete conditions, converts to a cytosolic aconitase [20, 21]. With growing evidence of HIF-2α as the master regulator of erythropoiesis, a functional IRE sequence was detected in the 5′ untranslated region (UTR) of HIF-2α mRNA [22–24]. Further in vivo studies established IRP1 as a principal regulator of translational de-repression of HIF-2α mRNA in iron-deficient cells, leading to its accumulation and increased stimulation of EPO expression [22, 25]. Furthermore, recent evidence has provided a foundation for studies to investigate the role of IRP1 and IRP2 in cancer biology [26, 27]. In this study, we found for the first time an association between a somatic splicing site *IRP1* mutation and PPGL-polycythemia syndrome.

## Case presentation

**Fig. 1:**
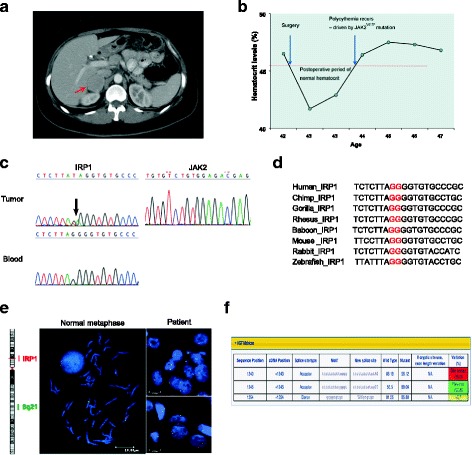
**a** Computed tomography (CT) demonstrating a right adrenal mass measuring 3.7 × 3.6 cm located between the liver and the upper pole of the right kidney. The left side shows a normal Y-shaped adrenal gland. **b** Marked in red is the upper reference limit for hematocrit levels. Patient had elevated hematocrit levels prior to the surgery and the hematocrit remained within the normal reference range for over a year after the surgery. **c** Genetic testing of the genomic DNA revealed a heterozygous mutation (c.267-1_267delGGinsTA; arrows) of the IRP1 gene in tumor, but not in blood. The JAK2 mutation was not detected in the patient’s tumor. **d** Alignment of base pair sequences of IRP1 in human, chimp, gorilla, rhesus, baboon, mouse, rabbit, and zebrafish, indicating its vital role in gene splicing. **e**. FISH analysis showed IRP1 deletion during metaphase in the tumor cells. Centromeric marker (green) and probe (red) are specific markers for chromosome 9 and IRP1. **f** Effects of mutations around splicing sites were predicted by Human Splicing Finder (HSF)

In 2013, a 44-year-old woman with a history of *JAK2*^V617F^ positive polycythemia vera was referred to the National Institutes of Health due to history of a right-sided pheochromocytoma, diagnosed and resected at age 42. The patient had been diagnosed with absolute erythrocytosis of unknown etiology at age 37 after presenting with classic clinical symptoms of aquagenic pruritus, facial plethora, excessive sweating, joint pain, fatigue and headache. Initially she had been treated with recurrent phlebotomies, and it was not until 2013 that *JAK2* genetic testing was performed. In 2011, she developed recurrent episodes of elevated blood pressure, palpitations, diaphoresis, abdominal pain, nausea and vomiting. Initial diagnostic workup revealed elevated levels of urinary norepinephrine, dopamine and normetanephrine. Subsequently, a 4.5 cm lesion was identified in the right adrenal gland on computed tomography (CT) (Fig. 1a).

The patient underwent an uneventful surgical resection of this mass, confirmed to be pheochromocytoma on pathology. Per patient, her polycythemia symptoms and hematocrit levels, continued to worsen over the years from 2007 to 2011, with an increased frequency of phlebotomies. Postoperatively, until early 2013, the patient’s hematocrit levels remained within the normal reference range and she did not require phlebotomies. EPO levels were not investigated prior to surgery and remained within the normal reference range postoperatively. The patient later developed mildly elevated hematocrit levels ranging between 46.4 and 48.9% [35–45%] with EPO in the normal reference range, requiring phlebotomies every 3–4 months (Fig. 1b). Serum iron, total iron binding capacity (TIBC), transferrin and ferritin results did not demonstrate iron deficiency. Additionally, recent testing in 2017 revealed only 2.11% of *JAK2*^V617F^ mutated clone in whole blood DNA. Of note, patient’s family history in the mother is significant for multiple thrombotic episodes of unclear etiology that led to a lethal pulmonary embolism despite being on prophylactic doses of warfarin. Laboratory and genetic testing results from the mother are not available.

Herein, the blood DNA from the patient was screened using our 54-gene panel, and no pathogenic mutations were detected. In tumor tissue, we identified an indel mutation of *IRP1* (c.267-1_267delGGinsTA) at the exon 3 splicing site, which is not present in the patient’s blood DNA (Fig. 1c). No mutations were detected in *HIF2A*, *PHD1/2* and the *VHL* gene in tumor specimen. The exon 3 splicing site of the IRP1 gene was highly conserved on multiple sequence alignment, indicating that this splicing site plays a vital role in IRP1 gene splicing (Fig. 1d). FISH demonstrated loss-of-heterozygosity (LOH) of *IRP1* in chromosome nine in the patient’s tumor tissue (Fig. 1e). The splicing site prediction algorithm (Human Splicing Finder) showed that the *IRP1* mutation either cause splicing site breakage or form a new splicing site, suggesting loss-of-function of the IRP1 protein (Fig. 1f).

**Fig. 2:**
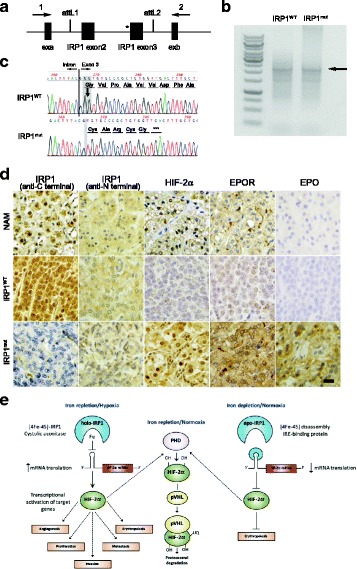
**a** Wild-type and mutant IRP1 were inserted into the exon trapping vector pSplice Express using the attL1 and attL2 sites. The inserted fragment is flanked by exon a (exa) and exon b (exb), which are two constitutive insulin exons from rat. **b** PCR products (indicated by arrow) were amplified from cDNA generated from Hela cells using primer 1 and 2, to analyze the splicing reporter. **c** Sanger sequencing results of cDNA generated from mutant minigene showed a frameshift mutation and premature stop codon. Wild type minigene was sequenced as the control. **d** Immunohistochemistry showed negative staining for IRP1 at C-terminal and positive staining for IRP1 at N-terminal, HIF2α, EPOR and EPO in tumor cells. Normal adrenal medulla (NAM) were used as normal control, and sporadic PHEO tumor was used as the wild type tumor control. **e** Regulation of erythropoiesis via IRP1/HIF2α/EPO pathway

To further confirm the effects of this mutation on splicing efficiency, we constructed minigenes consisting of exon 2 and 3 and intron 2, using the exon trapping vector (Fig. 2a). In the minigene and splicing site assay, the mutant minigene did not produce an exon skip transcript (Fig. 2b). Sanger sequencing of PCR products from the wild type and mutant minigenes demonstrated that the mutant minigene has two base pair deletion in exon 3, which cause a frameshift mutation from exon 3. Furthermore, the frameshift mutation led to a premature stop codon and truncated protein (p.V95*), causing a loss of 794 amino acids (Fig. 2c). Immunohistochemistry revealed that an anti-C terminal antibody of IRP1 could not detect the protein in patient tumor, while anti-N terminal antibody could bind to the protein, indicating that the protein was truncated as a consequence of the *IRP1* mutation, consistent with the sequencing results of minigene. HIF2α, which is negatively regulated by *IRP1,* was increased in the patient’s tumor tissue, compared to the normal adrenal medulla and wild-type IRP1 pheochromocytoma tissue. EPOR protein expression and EPO level were notably increased in the patient’s tumor tissue as well (Fig. 2d).

## Discussion and conclusions

The present study reports the first evidence of a novel somatic *IRP1* mutation associated with the pathogenesis of PPGL-polycythemia syndrome, further extending its genetic spectrum. This splicing site mutation causes a frame shift of exon 3, resulting in a heterozygous *IRP1* deletion and substantially decreased IRP1 protein levels in the tumor cells. As a result, due to inhibition of translational suppression of HIF-2α mRNA by IRP1, increased EPO expression is observed in tumor cells [22, 28]. These findings explain the exacerbation and transient resolution of secondary polycythemia before and after the resection of an erythropoietin-secreting PPGL, respectively, in a patient with *JAK2*^V617F^ positive polycythemia vera [29]. It is interesting that the expression of EPOR, which is induced by hypoxia [30] is also increased.

IRP1 and two are ubiquitously expressed cytosolic proteins, which share 56% sequence identity and display partial functional redundancy, regulated by different mechanisms [31]. A lack of both *IRP1* and *IRP2* alleles results in murine embryonic lethality at the blastocyst stage, supporting the crucial role of IRP/IRE machinery in early development [32]. In contrast, IRP1^−/−^ and IRP2^−/−^ mice are viable and exhibit distinct phenotypes. IRP1^−/−^ mice exhibit hyperproduction of EPO, resulting in extramedullary erythropoiesis and secondary polycythemia [18, 22, 28]. This is attributed to IRP1-regulated translational derepression of HIF-2α mRNA, which leads to upregulation of the hypoxia signaling pathway and induction of *EPO*, a target gene of HIF-2α (Fig. 2e) [18, 22, 24, 28].

HIF-2α is a transcription factor, encoded by *HIF2A/EPAS1*, which functions as an iron and oxygen- dependent master regulator of erythropoiesis [33]. HIF-2α mRNA harbors an IRE in its 5’ UTR, serving as a binding site for IRPs [23, 24]. IRP1 is a bifunctional protein, which, under iron replete conditions, assembles an aconitase-type 4Fe-4S cluster in its active site (referred to as holo-IRP1) and precludes binding to the HIF-2α mRNA IRE [20, 21, 34, 35]. In contrast, iron depletion promotes 4Fe-4S cluster disassembly and the conversion of holo-IRP1 to apo-IRP1, which binds to the HIF-2α mRNA IRE and represses its translation [36]. This homeostatic regulation acts as a safeguard mechanism to prevent erythropoiesis when there is reduced iron availability and vice versa. Furthermore, it has been proposed that hypoxia and normoxia regulate IRP1 binding to HIF-2α mRNA IRE, mimicking iron replete and deplete conditions, respectively [22, 37]. Under hypoxic conditions, holo-IRP1 is stabilized and HIF-2α mRNA is derepressed, resulting in upregulation of the hypoxia signaling pathway and increased production of EPO [25].

The previously described phenotype of IRP1 knockout mice is reminiscent of polycythemia associated with *VHL*, *PHD1/2* and *HIF2*A mutations. These mutations prevent proteasomal degradation of HIF-2α following iron and oxygen-dependent hydroxylation by PHDs and recognition by the pVHL E3 ubiquitin ligase [9, 11–16]. With the foundation of translational derepression of HIF-2α mRNA by IRP1 resulting in elevated erythropoietin levels and polycythemia, it may be hypothesized that this mechanism could also potentially activate other HIF-2α target genes involved in cancer development, progression and metastasis. While *IRP1*-mediated secondary polycythemia has never earlier been reported in humans, a single nucleotide polymorphism in *IRP1* was found to be associated with cutaneous malignant melanoma [29]. In addition, a previous study demonstrated suppressed tumor xenograft growth in nude mice transplanted with IRP1-transfected HI299 lung cancer cells [26]. Besides the association between HIF-2α mRNA and IRP1 in tumor pathogenesis, these studies provide an independent regulatory link between IRE/IRP system and cancer biology. This direct link is attributed to the crucial role of IRPs in iron homeostasis and how its disruption can cause chronic oxidative stress, resulting in malignant transformation and increased proliferation of neoplastic cells.

Our study provides the first evidence of a novel somatic *IRP* mutation in the pathogenesis of both cancer development and secondary polycythemia. One may challenge that the erythrocytosis in our patient is only due to the *JAK2*^V617F^ mutation since we do not have evidence of preoperatively elevated EPO levels. However, the *JAK2*^V617F^ mutated clone is only 2.11% and supports the observations of a very slightly raised hematocrit in our patient. Additionally, the upregulation of EPO and EPOR in the tumor tissue explains the worsening of erythrocytosis a few years prior to PPGL diagnosis and its transient resolution after resection of the tumor. Furthermore, EPO levels have been measured postoperatively since the patient’s first presentation to our institution and have remained within the normal reference range. These findings provide strong support that the polycythemia was driven by *IRP1*-induced erythrocytosis and the *JAK2* mutation is a minor contributor to the overall phenotype.

In conclusion, this novel loss-of-function mutation at the *IRP1* splicing site is the latest genetic abnormality to contribute to the pathogenetic factors associated with PPGL-polycythemia syndrome. Our study provides evidence to support the role of the IRP1-HIF-2α-EPO pathway in the pathogenesis of erythropoietin producing PPGLs. Testing for this genetic mutation could be considered in patients with evidence of an erythropoietin producing PPGL when other known HIF2A and PHD1/2 mutations have been ruled out.

## Abbreviations

CT: Computed tomography
EPO: Erythropoietin
EPOR: Erythropoietin receptors
FISH: Fluorescence in situ hybridization
HIF2A: Hypoxia-inducible factor type 2A
IRE: Iron-responsive element
IRPs: Iron regulatory proteins
PHD: Prolyl hydroxylase domain
PPGL: Pheochromocytoma/paraganglioma
TIBC: Total iron binding capacity
UTR: Untranslated region
VHL: von Hippel-Lindau
